# Endofibrosis as a Causative Agent of the Peripheral Artery Disease: A Report of Two Cases for Professional Cyclists

**DOI:** 10.7759/cureus.31406

**Published:** 2022-11-12

**Authors:** Tereza Mazurová, Ilker Sengul, Daniel Toman, Anton Pelikán, Demet Sengul, Miloslav Mazur, Petr Vávra, Václav Procházka

**Affiliations:** 1 Surgery, AGEL Ostrava Vitkovice Hospital, Ostrava, CZE; 2 Surgery, Ostrava University Faculty of Medicine, Ostrava, CZE; 3 Endocrine Surgery, Giresun University Faculty of Medicine, Giresun, TUR; 4 Surgery, Giresun University Faculty of Medicine, Giresun, CZE; 5 Surgery, University Hospital Ostrava, Ostrava, CZE; 6 Health Care Sciences, Tomas Bata University in Zlin Faculty of Humanities, Zlín, CZE; 7 Pathology, Giresun University Faculty of Medicine, Giresun, TUR; 8 Radiology, Ostrava University Faculty of Medicine, Ostrava, CZE; 9 Radiology, University Hospital Ostrava, Ostrava, CZE

**Keywords:** histopathology, vascular pathology, professional, cyclists, athletes, compression syndrome, iliac artery stenosis, endofibrosis, iliac artery, peripheral artery

## Abstract

Endofibrosis is a rare disease that predominantly affects athletes and is caused by a gradual occlusion of the (usually iliac) artery due to a thickening of the intima. From our experience, we report in this article two cases with the entity of endofibrosis in females around 30 years old. The first case presented with acute limb ischemia, and the second one was with pain in the leg during exercise. In addition, both cases are professional cyclists. They were eventually diagnosed with endofibrosis and underwent surgical procedures. They are now pursuing their professional career successfully. Last but not least, endofibrosis might be classified as an occupational disease, particularly, in the case of professional athletes or cyclists.

## Introduction

Endofibrosis is a rare condition, which affects blood vessels and occurs mainly among young, otherwise healthy athletes. This condition arises as progressive stenosis of the iliac arteries, which attenuates the blood circulation of the limb, thus causing pain, weakness, or numbness during movement and reducing the performance of the cases [1]. Iliac artery compression was first described in 1984 by professional cyclists [2]. Stenosis often occurs due to anatomical, mechanical, and postural causes. Repeated hip hyperflexion leads to trauma of the vessel wall and hypertrophy of the psoas muscle. The psoas muscle then compresses the artery and causes stenosis [3]. Endofibrosis is one of the rare causes of peripheral artery disease (PAD). More than 90% of PAD is caused by atherosclerosis. However, the exact prevalence of endofibrosis is unknown, and there is no data for this, but it is less than 0.01%. Due to demanding physical activities, athletes are initially considered to suffer from a neurological or musculoskeletal problem, which causes a delay in diagnosis. As the awareness of endofibrosis is insufficient, the diagnosis is often late [4]. The detailed personal history, symptoms, low ankle-brachial index (ABI), and Doppler sonography of the lower limb arteries (possibly augmented with other imaging techniques) are essential for the correct diagnosis of endofibrosis according to the 2016 Delphi consensus [5].

## Case presentation

Two vignette cases with endofibrosis had been managed at the Department of Surgery, Nemocnice AGEL Ostrava-Vítkovice, Ostrava, Czech Republic, from 2017 to 2018. Both of them were professional cyclists.

Case 1

The first case was that of a female aged 29 years, who presented with no medical history, and the symptoms such as pain in the right leg, numbness, and paresthesia in rest occurred suddenly. As a result, the anticoagulant treatment had been administrated to her, and the symptoms disappeared with emerging physical stress. The patient was diagnosed with an embolism. During further diagnosis, the ABI, Doppler sonography, and computed tomography angiography (CTAG) had been performed. The rest ABI index was found to be in the normal range of 1.15, but it decreased to 0.48 after exercise. The colored Doppler ultrasonography (CDU) and CTAG revealed the total occlusion of the external iliac artery (EIA), and endofibrosis was diagnosed based on the history of the patient. Due to the patient's desire to return to sports, surgical management was chosen. We performed deliberation of the EIA from the hypertrophic psoas muscle, thrombectomy, endofibrosectomy, and venous patch from the great saphenous vein (GSV). Histopathologically, there was a part of the arterial wall where the endothelium was largely preserved, quite rarely eroded with fibrinous thrombus, subendothelial with the accumulated, relatively few cell connective tissue, which was focally edematous, only sporadically proliferating, and partly fibrotic. The media appeared to be within the norm, without compelling hypertrophy. The patient was monitored for one day in the intensive care unit (ICU) and one day in the inpatient clinic of the Department of Surgery. The postoperative sonography exhibited a normal triphasic waveform. She was discharged on the third postoperative day and returned to cycling within two months.

Case 2

**Figure 1:**
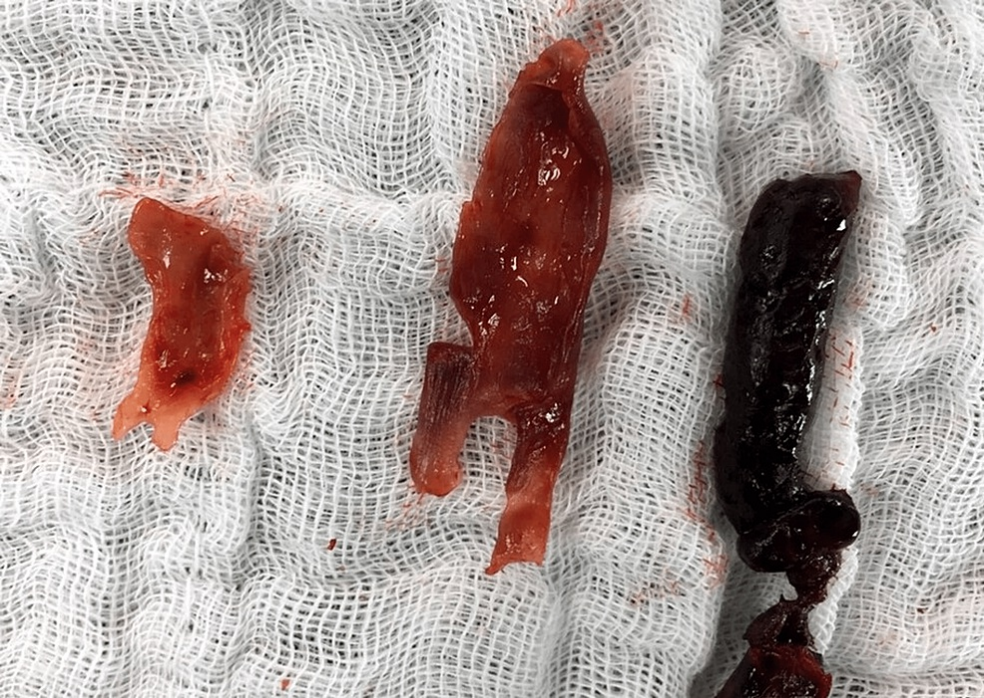
The postoperative photograph: the thrombectomy (thrombus) and endofibrosectomy

**Figure 2:**
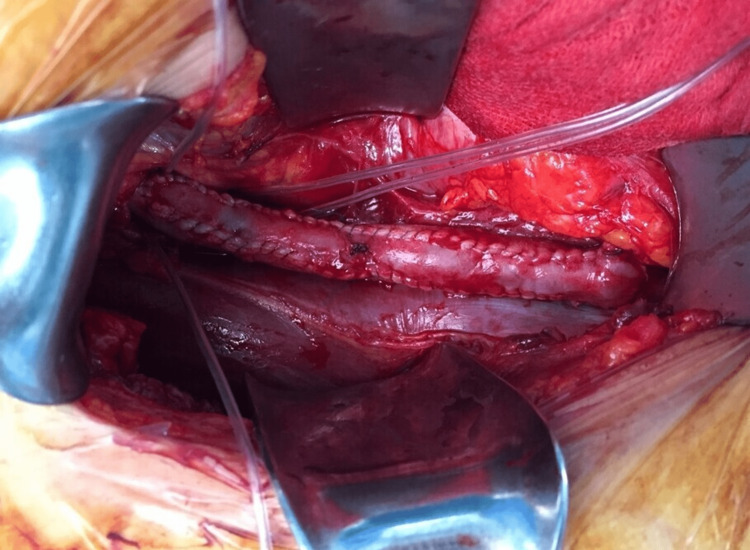
The intraoperative photograph: the patch venosus and the great saphenous vein graft

The second case was that of a female aged 31 years, who presented with post-exercise pain in the left leg. She was referred to our center for further examination and management. The rest ABI index was detected at the normal range (1.00), but it decreased to 0.42 after exercise. The CDU exhibited a normal triphasic waveform at rest but pathological monophasic waveforms after exercise with stenosis of the EIA. The stenosis of the EIA was recognized according to the CTAG. The diagnosis of endofibrosis was established, and the patient underwent a surgical procedure, including deliberation of the EIA from the hypertrophic psoas muscle, endofibrosectomy, and venous patch from the GSV (Figures 1, 2). The patient was monitored for one day in the ICU and one day in the inpatient clinic of the Department of Surgery. The postoperative ultrasound exhibited normal triphasic waveforms, and she was discharged on the third postoperative day. She came back to professional cycling activity in six weeks.

The patients are still professional cyclists with no limitations after 49 and 30 months of follow-up, and the primary patency is 100%. Normal triphasic waveforms were observed in postoperative ultrasounds at six weeks and three, six, 12, 24, and 36 months.

## Discussion

**Figure 3:**
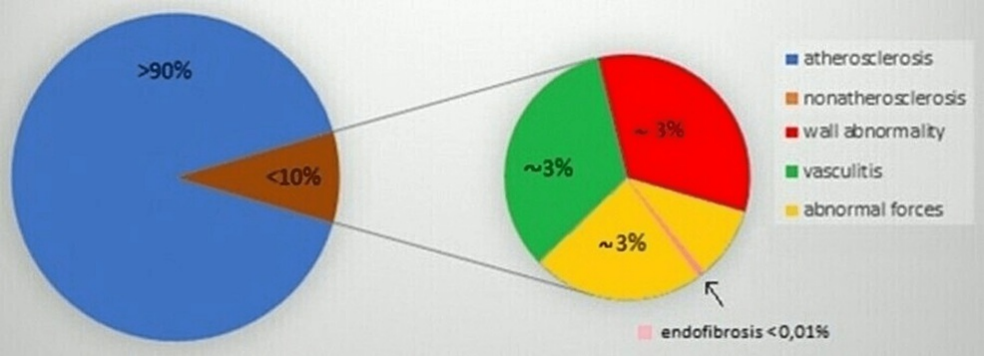
Causative agents of peripheral artery disease

Endofibrosis is one of the rare causes of PAD. More than 90% of PAD is caused by atherosclerosis. However, the exact prevalence of endofibrosis is unknown as there is no data for this, but it is less than 0.01% (Figure 3). Endofibrosis, per se, is described as a regeneration process that is exhibited as a progressive disease. In addition, the regeneration process remains controversial, particularly with the cases usually being annoyed for unnecessarily long time intervals [6]. Iliac endofibrosis should always be considered as a possibility in the case of claudication in athletes, particularly cyclists. Early diagnosis will attenuate unnecessary examinations, prevent disease progression, and offer early treatment. CDU is the imaging method of choice in diagnosing endofibrosis, mostly due to its non-invasive nature and high sensitivity of up to 85% [7]. Nevertheless, there are no official guidelines on how to treat a patient with endofibrosis. According to the available literature, there are a few options. If a professional athlete is diagnosed with endofibrosis, he/she should undergo surgery. If the patient is not a professional athlete but is very limited in his life, he/she should undergo surgery too. If there is no big limitation in life, the patient should stop the provocative activity and follow the rules for atherosclerosis risk reduction. However, endovascular therapy has no place in the treatment of endofibrosis [8-11].

We suggest that patients who are not top athletes should start with conservative management. They should be advised to abstain from exercise causing difficulty while undertaking other sporting activities, thus reducing the risk factors of atherosclerosis. On the other hand, surgical treatment should be a primary recommendation for professional athletes [11]. Nevertheless, a conservative approach might be recommended for the other risk groups [8-12].

## Conclusions

So far, there are no complete guidelines to follow, which, among other things, leads to relatively late diagnosis. As endofibrosis is a progressive disease, patients often suffer for unnecessarily long time intervals. It is also worth considering that endofibrosis should be classified as an occupational disease in the case of professional athletes or cyclists. In the cases presented in this article, we would like to demonstrate the use of surgical procedures that agree with the up-to-date literature knowledge in two patients. The treatment in both cases was revealed to be highly successful, especially when the patients returned to their professional athletic performances in their difficult but good jobs.
